# Immunogenicity and safety of inactivated quadrivalent influenza vaccine compared with the trivalent vaccine for influenza infection: an overview of systematic reviews

**DOI:** 10.1186/s12879-023-08541-0

**Published:** 2023-08-29

**Authors:** Rodrigo Luiz Carregaro, Alessandra N. C. P. Roscani, Augusto Cesar Sousa Raimundo, Larissa Ferreira, Tazio Vanni, Maria da Graça Salomão, Livia Fernandes Probst, Juliana Yukari K. Viscondi

**Affiliations:** 1 Center for Evidence and Health Technology Assessment (NETecS), Universidade de Brasília (UnB), Campus UnB Ceilândia, Centro Metropolitano, Ceilândia Sul, CEP: 72220-275 Brasília/DF Brazil; 2 Universidade de Campinas (UNICAMP), Clinical Hospital Unity, Campinas, Brasil; 3 Faculty of Dentistry, Universidade de Campinas (UNICAMP), Piracicaba, Brasil; 4 Institute of Health Strategy Management of the Federal District, Department of Health of the Federal District (SES/DF), Brasília, Brazil; 5 Instituto Butanta, São Paulo, Brazil; 6 Health Technology Assessment Unit, MBA in Health Technology Assessment, Oswaldo Cruz German Hospital (HAOC), São Paulo, Brazil; 7 Hospital de Base, Secretaria de Estado de Saúde do Distrito Federal, Brasília, Brazil

**Keywords:** Flu, Vaccination, Efficacy, Immunogenicity

## Abstract

**Background:**

Influenza infection is a highly preventable transmissible viral disease associated with mild upper respiratory symptoms and more severe conditions such as lethal pneumonia. Studies have shown that a broader spectrum influenza vaccine could reduce influenza’s burden of disease in low- and middle-income countries. A considerable number of systematic reviews reported that quadrivalent influenza vaccines are considered more effective compared to trivalent vaccines, hence, there is a need for an overview in order to synthesize the current evidence pertaining to the comparison between quadrivalent and trivalent inactivated influenza vaccines. Objective: The aim was to summarize the evidence from systematic reviews that investigated the immunogenicity and safety of the Influenza’s inactivated quadrivalent vaccine (QIV) compared to the trivalent vaccine (TIV), in the general population.

**Methods:**

We searched articles up to December 2022 at: Web of Science, EMBASE, MEDLINE, Cochrane Library, and SCOPUS. The search strategy was conducted following the PICO model. We included systematic reviews comparing the primary outcomes of immunogenicity (seroprotection rate and seroconversion rate) and adverse events using risk ratios. The AMSTAR 2 and ROBIS were used for quality assessments, and GRADE was used for evidence certainty assessments.

**Findings:**

We included five systematic reviews, totalling 47,740 participants. The Quadrivalent Inactivated Influenza Vaccine (QIV) exhibited enhanced immunogenicity in the context of B-lineage mismatch when compared to the Trivalent Inactivated Influenza Vaccine (TIV). While the safety profile of QIV was found to be comparable to that of TIV, the QIV showed a higher incidence of solicited local pain among children and adolescents, as well as an increased frequency of local adverse events within the adult population.

**Conclusion:**

Our findings suggest that the QIV provides a superior immunogenicity response compared to the TIV in all age groups evaluated, especially when a lineage mismatch occurred. The safety of QIV was considered similar to the TIV, with no serious or systemic solicited or unsolicited adverse events; tough pain at the injection site was greater for QIV. We recommend caution owing to the high risk of bias in the selection process and no protocol registration.

**Supplementary Information:**

The online version contains supplementary material available at 10.1186/s12879-023-08541-0.

## Background

It has long been recognized that influenza is a highly contagious viral disease that can be effectively prevented. This disease is primarily characterized by mild upper respiratory symptoms, fever, headache, and muscle fatigue. However, it is important to note that influenza can also lead to more severe complications, such as life-threatening pneumonia, particularly among children and elderly individuals. Influenza infections are known to occur annually during seasonal epidemics. Nevertheless, owing to the considerable variability of the influenza virus, sporadic and unpredictable pandemic outbreaks can emerge intermittently, with intervals spanning from 10 to 50 years [1]. Currently, there are four main of human influenza lineages circulating: A/H1N1, A/H3N2, B/Victoria, and B/Yamagata. While Influenza A and B have similar symptoms, Influenza B accounts for an estimated 15% of all respiratory- and circulatory-related deaths attributed to influenza in the US [2]. Influenza B affects individuals of all age groups, with a higher incidence relative to influenza A among children and young adults. For instance, a previous study showed that the proportion of illness caused by influenza B was considered higher on school-aged children (i.e., 5 to 17 years) [3].

Influenza surveillance studies in different countries [4–7] have shown that during mismatch seasons, there is a higher number of influenza B infections (B/Yamagata-like and B/Victoria-like) compared to non-mismatch years. This increase in cases of influenza B infections during mismatch seasons has the potential to lead to more severe disease. Additionally, a previous study reported that Influenza B lineages prevailed in eight seasons in a tertiary hospital between 2001 and 2013 [4], although B/Yamagata-like strains and B/Victoria-like strains varied consistently within seasons. However, there are “mixed” seasons in which both B lineages did not vary [4, 8], and the mismatch between the trivalent vaccine lineage and the predominant lineage occurred in approximately one third of the seasons [8]. In addition, a recent study reported that the proportion of influenza B vaccine mismatch was around 54% in Southern hemisphere countries, and 43% in Northern countries [9]. Moreover, comparisons considering the B lineage included in trivalent influenza vaccine (TIV) and the circulating B lineages have demonstrated that more than 50% of influenza B viruses belonged to lineages not included in the seasonal TIV [10]. Therefore, the unpredictability circulation of Influenza B lineages may pose a burden and increase the risk of severe conditions during a vaccine mismatch season.

The most practical way to prevent influenza is improving the population’s immunologic responses through vaccination [11]. Since the 1980’s, a trivalent vaccine (including both A strains and one B-lineage) has been introduced worldwide [12]. However, despite careful selection of the B strain included in the trivalent vaccine for each seasonal epidemic, there is a recurrent mismatch [8, 13] that could compromise the vaccine’s effectiveness and, consequently, the prevention strategies of healthcare systems.

Studies have shown that a broader spectrum influenza vaccine may reduce the burden of influenza in low- and middle-income countries [14–16]. Several systematic reviews have reported that quadrivalent influenza vaccines were considered more effective than trivalent vaccines, particularly during the mismatch season [17–26]. While quadrivalent influenza vaccines offer broader protection against influenza B disease, there is still significant debate regarding the advantages and disadvantages of using trivalent versus quadrivalent vaccines in combating influenza and preventing influenza epidemics [11]. Moreover, a recent review [15] demonstrated that although the quadrivalent influenza vaccine tends to be more effective and cost-effective compared with the trivalent vaccine, vaccination coverage and resource constraints and low- and middle-income countries may affect the outcomes of vaccination programs. Therefore, there is a need for an overview of systematic reviews in order to synthesize the current evidence comparing quadrivalent and trivalent inactivated influenza vaccines.

The aim of this study is to provide a summary of the evidence from systematic reviews that have examined the immunogenicity and safety of inactivated quadrivalent influenza vaccines, in comparison to trivalent vaccines, among the general population.

## Method

To address the question “*Is the inactivated quadrivalent influenza vaccine more immunogenic and safer compared to the trivalent vaccine?*”, we conducted an overview of systematic reviews. This overview was prospectively registered in PROSPERO (CRD: 42,022,309,321). The research process followed the recommendations outlined in Chapter V - Overviews of Reviews of the Cochrane Handbook for Systematic Reviews of Interventions [27]. The PRIOR (Preferred Reporting Items for Overviews of Reviews) statement was used to report this study [28].

### Eligibility criteria

We established eligibility criteria for the inclusion of studies using the PICO model. The PICO acronym and its details are presented in Table 1.

The reviews were included if they met the following criteria: (1) Investigated Influenza; (2) Adopted inactivated vaccine administered intramuscularly; (3) Included human participants; (4) Consisted exclusively of systematic reviews that encompassed either randomized clinical trials or non-randomized clinical studies. We decided to investigate inactivated vaccines due to the fact that this type of vaccine is the most used worldwide.

The exclusion criteria for this study were as follows: (1) Studies comparing quadrivalent vaccine with a placebo or any vaccine other than trivalent; (2) Studies involving immunocompromised participants.

**Table 1:** Study characteristics used as eligibility criteria

**Population**	All age groups from the general population (> 6 months).
**Intervention**	Quadrivalent inactivated influenza vaccine (inactivated fragmented without adjuvant) via intramuscular application.
**Comparator**	Trivalent inactivated influenza vaccine (inactivated fragmented without adjuvant) via intramuscular application.
**Outcomes**	Primary outcomes: 1) Immunogenicity of Influenza B lineages: measured by the seroconversion rate (SCR), seroprotection rate (SPR), and geometric mean titer ratio (GMTR). 2) Safety: measured by the number of Adverse Events (AEs) and Serious Adverse Events (SAEs) following vaccination. Secondary outcomes: • Number of cases of laboratory-confirmed Influenza Like Illness. • Number of laboratory-confirmed cases of SARI (Severe acute respiratory infection). • Number of hospitalizations due to influenza. • Number of laboratory-confirmed cases of pneumonia. • Number of laboratory-confirmed cases of acute otitis media. • Number of cases of mortality due to influenza. Exploratory outcomes: • Quality-Adjusted Live Years (QALY). • Work days lost.

### Information sources

An electronic search was conducted in the following databases, from their inception to December 2022: Web of Science (1945–2022), EMBASE (Excerpta Medica Database, 1947–2022), MEDLINE via PUBMED (National Library of Medicine, 1950–2022), CINAHL (Cumulative Index to Nursing and Allied Health Literature, 1982–2022), LILACS (Latin American and Caribbean Health Science, 1982–2022), Cochrane Library (1988–2022), and SCOPUS (1996–2022).

### Search strategy

The search strategy involved combining medical subject headings according to the PICO model. The detailed search strategies are presented in detail in the Appendix Table 1. An experienced librarian validated the electronic search strategy. The search was not restricted to any language or publication date. We also examined the reference lists of the included reviews and the PROSPERO registry database to identify potential studies that which may not have been found through the search strategy. The results of the search strategy were also validated within the Epistemonikos database [29].

### Selection process

The results from each database were uploaded into the COVIDENCE platform [30] to control duplicates and extract further information. Two independent reviewers (A.P.R and L.F.) screened all titles and abstracts for relevance. The same reviewers examined the full texts to determine which studies to include. In cases of disagreement, a third reviewer was consulted to resolve discrepancies.

### Data collection process

After the full-text screening, a specific form was used to extract the following information of the included reviews: authors/year, title, setting, study type, country, keywords, searched databases, number of studies included, participants, funding source, intervention, comparator, outcomes, quality assessment, and conflict of interest statements. One reviewer (A.P.R) extracted the descriptive characteristics of the methods, sample, intervention, and outcomes reported in each study. The second reviewer (L.F.) independently extracted the same data, and disagreements were resolved through discussion and consensus. We assessed the overlap between the included systematic reviews by quantifying the number of primary studies included and the corresponding number of participants. Subsequently, we compared the systematic reviews and noted which primary studies were duplicated.

### Data items

Primary outcomes presented in Table 1 were assessed using risk ratios (RR) and its 95% confidence interval (95%CI) as the summary measure. The immunogenicity was evaluated based on the number of participants presenting specific seroconversion rates (SCRs) and seroprotection rates (SPRs). SCRs were defined as the proportion of participants with hemagglutination inhibition antibody titer < 1:10 before vaccination and ≥ 1:40 after vaccinated (follow-up days) or > 1:10 before vaccination and a ≥ 4-fold increase in antibody titer after vaccinated (follow-up days). Additionally, SPRs were defined as the proportion of participants who attained seroprotection with an hemagglutination inhibition antibody titer ≥ 1:40 at the follow-up [17, 18, 20, 21].

Safety was assessed by the number of participants presenting solicited and unsolicited local (e.g., injection-site events such as pain, redness, swelling) and/or systemic adverse events (e.g., fever, irritability, drowsiness) caused by the vaccines. The follow-up period adopted by each study was taken into consideration.

If available, the secondary outcomes (Table 1) were assessed based on the number of events and, and the effect measures were risk ratios or odds ratios. Quality-Adjusted Live Years and workdays lost were evaluated based on mean differences.

### Methodological quality and risk of bias assessment

The methodological quality and risk of bias of the included reviews were assessed by other independent reviewers (A.C.S.R and R.L.C.). The Critical Appraisal Tool for Systematic Reviews that include randomized or non-randomized studies of healthcare interventions (AMSTAR 2) [31], and ROBIS (Risk Of Bias In Systematic Reviews) [32] were used for this purpose. Initially, we planned to use the ROBIS assessment specifically for reviews exclusively consisting of randomized controlled trials. However, all the included reviews in our overview ended up addressing only randomized clinical trials, so we applied ROBIS to all of them. Discrepancies in the appraisal were resolved through discussion to reach an agreement and consensus. In cases where questions arose or clarification was needed regarding pertinent information relevant to the quality assessment in any of the studies, the corresponding author was contacted by email. If none of the authors could be contacted or if the information was no longer available, the specific item was finally marked as “no” (absent) in AMSTAR 2, or “no information” in the ROBIS assessment.

Results from the quality assessment and risk of bias assessments were presented descriptively using tables. The impacts of each rating on AMSTAR 2 were discussed rather than creating an overall score [31]. We utilized the Risk of Bias Visualization tool (ROBVIS, available at: https://www.riskofbias.info/welcome/robvis-visualization-tool), to generate the risk of bias table of the included reviews.

### Evidence certainty assessment

The same independent reviewers (A.C.S.R. and R.L.C.) extracted the outcomes that had been investigated in the included reviews and assessed the quality of the evidence using the Grading of Recommendations, Assessment, Development, and Evaluation (GRADE) approach. The assessment was conducted following the GRADE instructions [33]. If the GRADE assessments were not available in the included studies, we assessed the quality of evidence using data reported in the systematic reviews, in accordance with the recommendations of Cochrane’s Handbook [27]. The reviewers made judgements independently, using a specific checklist developed for reviews of interventions [34]. The ‘Summary of findings’ and ‘Evidence profile’ tables using the GRADE tool (GRADEpro). The checklist [34] includes detailed questions for evaluating meta-analyses of randomized controlled trials to inform the GRADE assessment. The checklist covers the main determinants for each of the five GRADE assessment’s criteria: risk of bias, inconsistence, indirectness, imprecision and publication bias. The risk of bias domain was judged based on the assessments performed by the authors of the included reviews. All the included systematic reviews used the Cochrane Risk of Bias tool (ROB version 1.0). In cases of disagreement in the GRADE assessment, consensus was reached through discussion.

For the summary and evidence profile tables, we focused on the primary outcomes, considering the immunogenicity as an important outcome, and adverse events as a critical outcome with clinical relevance for patients. Furthermore, the main comparisons between the quadrivalent and trivalent vaccines regarding immunogenicity (seroconversion and seroprotection) were specifically addressed, taking into account the influenza vaccine’s B lineage mismatch.

## Results

### Systematic reviews selection

A total of 2,244 studies were identified through the database search. After removing duplicates, 1,159 records were screened. After reviewing the titles and abstracts, 1,135 studies were excluded, leaving 24 studies for further full-text appraisal. Finally, 19 studies did not meet the inclusion criteria and were excluded [19, 22, 35–51]. Appendix Table 2 provides details on the 19 excluded studies.

**Fig. 1:**
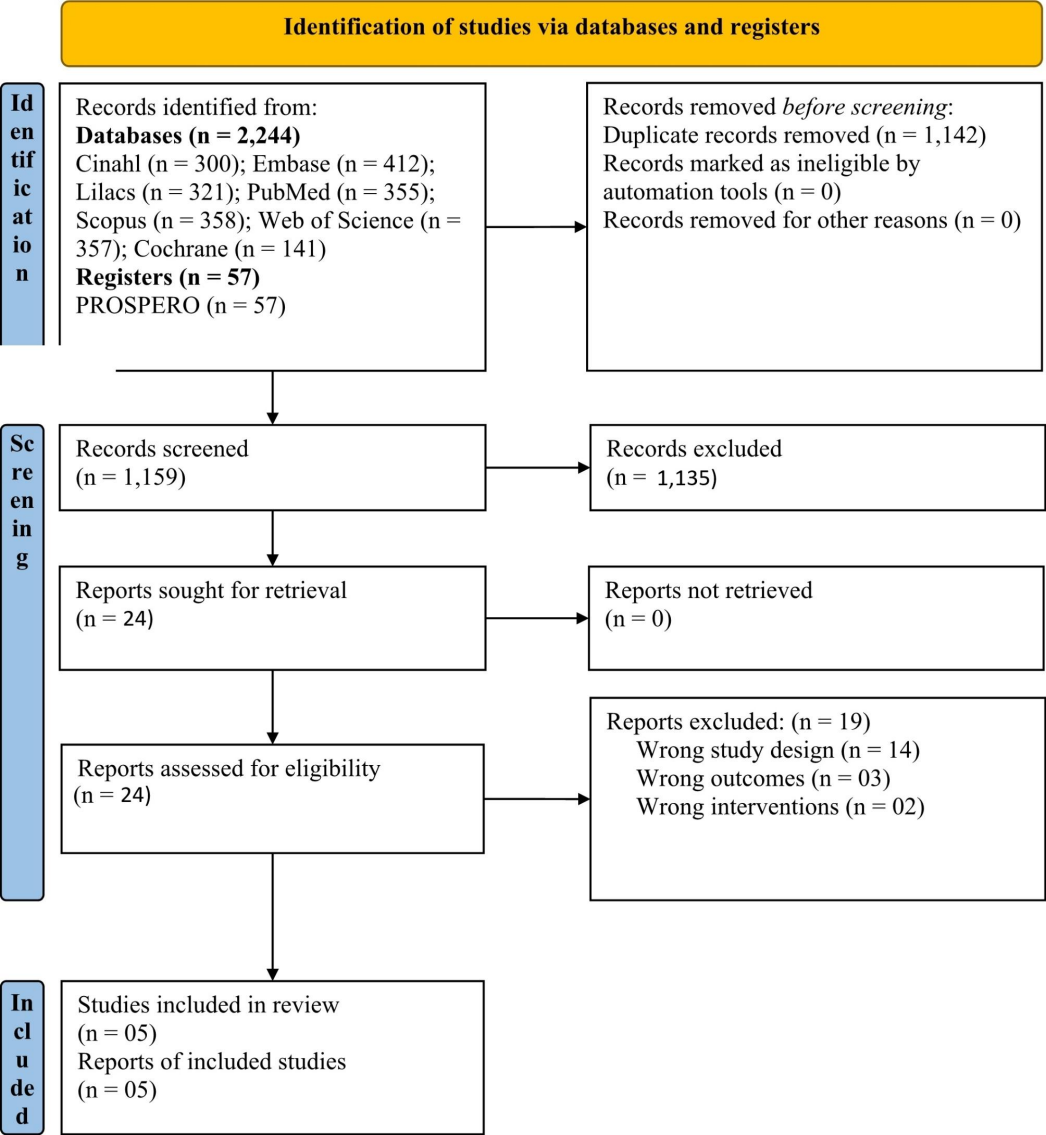
Study flowchart

Five systematic reviews [17, 18, 20, 21, 52] were included in this overview (see Fig. 1). The characteristics of the reviews are presented in Table 2. All five systematic reviews included randomized controlled trials only, totaling 53,896 participants (excluding duplicated primary studies) and investigated the outcomes immunogenicity and adverse events. Only one systematic review [52] presented data on the secondary outcomes, specifically the number of hospitalizations, number of cases of acute otitis media, and number of influenza-like illness.

### Characteristics of systematic reviews

The five reviews included in this overview focused randomized trials that recruited the general population, including children, adolescents, adults, and the elderly. The trials within these reviews were conducted in a wide range of countries, including middle- and high-income countries.

Table 2 provides details of the included reviews. The systematic reviews also provided relevant data pertaining to the analysis of immunogenicity for influenza B lineages. Eight comparisons were made for seroprotection and seroconversion in adults, while four comparisons were made for seroprotection and seroconversion in children and adolescents. The reviews also assessed local and systemic adverse events. (Table 2).

### Primary study overlap

A total of 56 primary studies were included in the systematic reviews. After removing duplicated records, the total number of primary studies was 30. 50% of the primary studies were included exclusively in one review. The extent of primary study overlaps across the included systematic reviews ranged from 10 to 26.7%. This means that some primary studies were duplicated in two reviews (26.7%), three reviews (10%), and four reviews (13.3%).

**Table 2:** Characteristics of the included studies

First Author, Published Year	Moa, 2016	Meng, 2018	Huang, 2020	Liang, 2021	Minozzi, 2022
Type of study	Systematic review	Systematic review	Systematic review	Systematic review	Systematic review and NMA
Country of primary studies	Australia, United States, Czech Republic, Germany, Romania, Spain, Korea, Taiwan, France, Canada and Mexico	China, United States, Czech Republic, Germany, Romania, Spain, Korea, Taiwan, France, Canada, Mexico, China, Belgium and Poland	China, United States, Canada, Dominican Republic, Honduras, Asia, Latin America, Europe, South Korea, China, Czech Republic, France, Germany, Philippines, Mexico, Spain, Taiwan, Poland, Finland	China, United States, Czech Republic, Germany, Romania, Spain, Korea, Taiwan, France, Canada, Mexico, China, Belgium and Poland	Continental Europe, Africa, Asia, North America, South America, Oceania, Multi-continent
Study period	6/30/2015	Not informed	2/12/2019	2011–2020	12/15/2020
Search strategy	Not informed	Not informed	(quadrivalent OR tetravalent) AND (influenza OR flu) AND vaccine	QIV and TIV	#1 “Influenza, Human”[MeSH] #2 “Influenzavirus A”[MeSH] #3 “Influenzavirus B”[MeSH] #4 influenza*[Text Word] OR flu[Text Word] #5 #1 OR #2 OR #3 OR #4 #6 “Vaccines”[MeSH] #7 “Immunization”[MeSH] #8 (vaccin*[Text Word] OR immuni*[Text Word] OR inocula*[Text Word]) #9 #6 OR #7 OR #8 #10 #5 AND #9 #11 “Influenza Vaccines”[MeSH] #12 #10 OR #11 #13 “Randomized Controlled Trial” [Publication Type] #14 “Controlled Clinical Trial” [Publication Type] #15 randomized[Title/Abstract] #16 placebo[Title/Abstract] #17 “drug therapy” [Subheading] #18 randomly[Title/Abstract] #19 trial[Title/Abstract] #20 groups[Title/Abstract] #21 #13 OR #14 OR #15 OR #16 OR #17 OR #18 OR #19 OR #20 #22 (“Animals”[MeSH]) NOT “Humans”[MeSH] #23 #21 NOT #22 #24 #12 AND #23
Searched databases	Medline, EMBASE, Cochrane Central Register of Controlled Trials, Scopus and Web of Science	Medline, Cochrane Library, Science Direct	PubMed, EMBASE and Cochrane Library	Cochrane Library, PubMed, ClinicalTrials.gov, EMBASE, China Biology Medicine disc, Chinese National Knowledge Infrastructure, and Wanfang Data.	Cochrane Central Register of Controlled Trials (CENTRAL) (The Cochrane Library), MEDLINE (PubMed) and EMBASE
Number of included studies, and phase	5 RCT (1 phase I/II, 1 phase II and 3 phase III)	8 RCT (1 not informed, 1 phase I/II, 1 phase II and 5 phase III)	9 RCT (3 phase II and 6 phase III)	9 RCT (1 not informed, 1 phase I/II, 1 phase II and 6 phase III)	220 RCT (phase not informed)
Population	Adults aged > 18 years	Adults between 18 to 64 years of age	Healthy children and adolescents aged from 6 months to 18 years	Adults between 18 to 64 years of age	Healthy children (< 18 years), healthy adults (18 − 60 years), and the elderly (age ≥ 61 years)
Total number of participants	8,934	15,123	14,819	16,422	429,804
Intervention	QIV vaccine, 15 μg haemagglutinins per strain, and were given as 0.5 mL dose intramuscularly.	Quadrivalent inactivated influenza vaccine (QIV)	QIV contained 15 μg each of the A/H1N1, A/H3N2, B/Victoria and B/Yamagata	Quadrivalent inactivated influenza vaccine (QIV)	Trivalent and quadrivalent inactivated influenza vaccines (IIVs) (whole virus, split or sub-unit) administered intramuscularly (IM) or intradermically (ID), live-attenuated influenza vaccines (LAIVs) administered by intranasal (IN) route, recombinant influenza vaccines (RIVs) administered IM.
Comparator	TIV vaccines, 15 μg haemagglutinins per strain, and were given as 0.5 mL dose intramuscularly.	Trivalent inactivated influenza vaccine (TIV)	TIV contained 15 μg each of the A/H1N1 and A/H3N2 and 15 μg of the B/Victoria or B/Yamagata	Trivalent inactivated influenza vaccine (TIV)	Trivalent and quadrivalent inactivated influenza vaccines (IIVs) (whole virus, split or sub-unit) administered intramuscularly (IM) or intradermically (ID), live-attenuated influenza vaccines (LAIVs) administered by intranasal (IN) route, recombinant influenza vaccines (RIVs) administered IM.
Quality assessment	ROB 1.0	ROB 1.0	ROB 1.0	ROB 1.0	ROB 1.0
Funding sources	Not informed	Not informed	National Natural Science Foundation of China and Mega-Project of National Science and Technology for the 12th and 13th Five-Year Plan of China	Medical Science and Technology Innovation Project of Chinese Academy of Medical Sciences, Medical Science and Technology Innovation Project of Chinese Academy of Medical Sciences and Innovation Team in Yunnan Province	Directorate general of welfare, Lombardy region.
Conflicts of interest	From one author that contributed to the study design, statistical analysis, and editing of manuscript.	No potential conflicts of interest were disclosed.	No potential conflicts of interest were disclosed.	No potential conflicts of interest were disclosed.	One author declares support for attending meeting and travel by Sanofi and Seqirus and, participation in Advisory Board of Sanofi. One author declares participation in Advisory Board of Sanofi.

RCT: Randomized Controlled Trial; ROB: Cochrane’s risk of bias; NMA: Network metanalysis.

### Methodological quality and risk of bias

Details on the AMSTAR 2 assessment are presented in Appendix Table 3. Four of the included reviews were classified as critically low quality [17, 18, 20, 21], and one review was judged as low quality [52]. This was mainly due to the absence of critical items, such as the study protocol or no information regarding the source of funding of the primary studies. Additionally, four reviews did not report the list of excluded studies with the reasons for exclusion [17, 18, 20, 21] and none of the reviews considered the impact of the risk of bias on the meta-analyses results. Furthermore, none of the systematic reviews investigated the publication bias.

The risk of bias assessment details can be found in Table 3. Overall, the ROBIS assessment of 4 reviews were classified as low risk of bias, and 1 as high risk [18]. However, four reviews [17, 18, 20, 21] were classified as having high risk of bias in the domain 2 (identification and selection of studies).

**Table 3:**
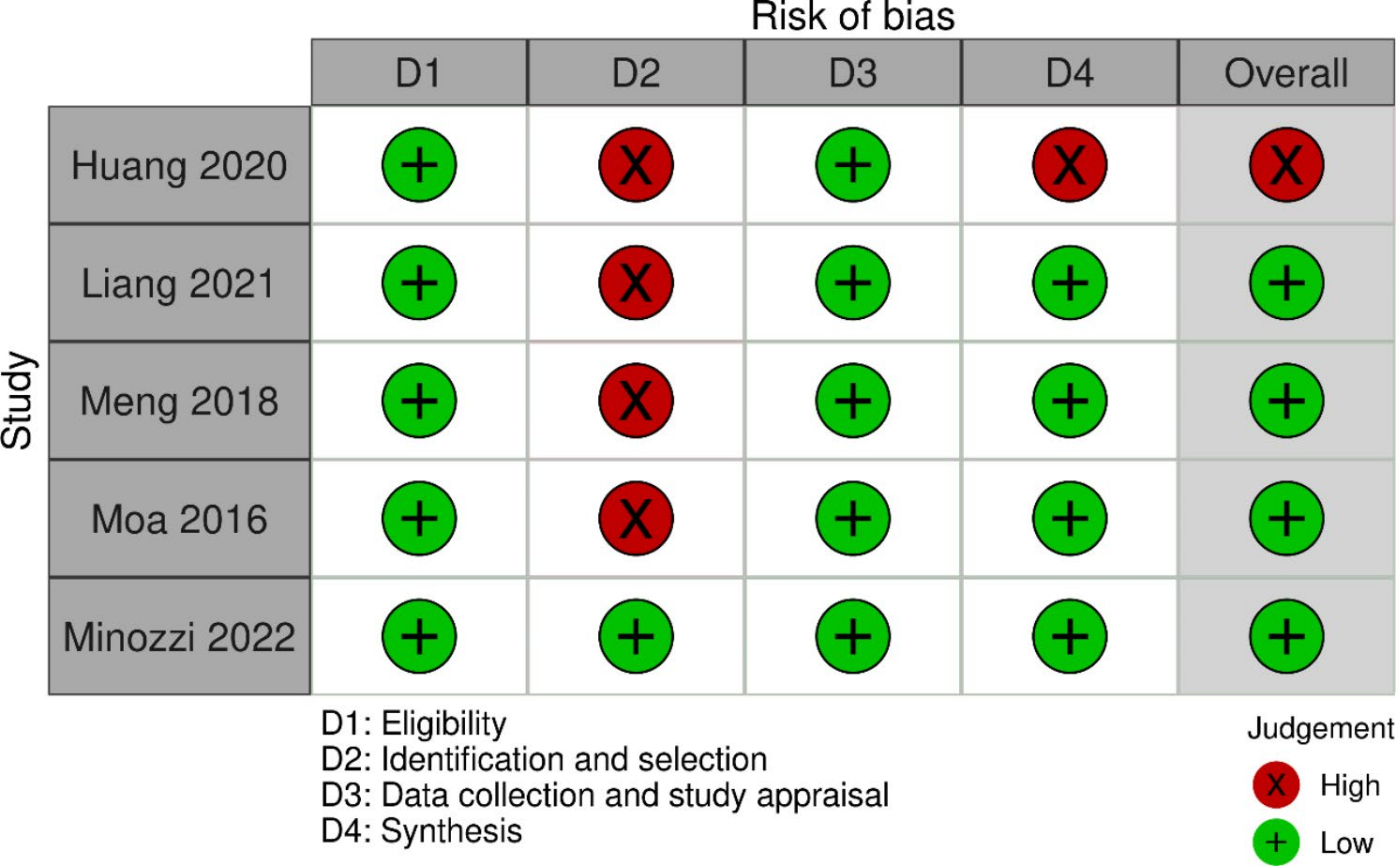
Data on the risk of bias assessment (ROBIS)

### Summary of results

Three of the included reviews provided data exclusively on seroconversion and seroprotection rates of QIV compared to TIV in the adult population [17, 20, 21]. One review presented data on seroconversion and seroprotection rates of the QIV versus TIV in children and adolescents [18]. Another review [52] provided data for QIV versus TIV on all age groups, including the number of hospitalizations, cases of acute otitis media in children, influenza-like illnesses, and adverse events, but not seroconversion and seroprotection rates. It is worth noting that Minozzi’s review [52] did not stratify for the lineage mismatch; thus, their results provided a pooled estimate. There were no data available on the other outcomes (i.e., laboratory-confirmed cases of pneumonia, severe acute respiratory infection, mortality, QALY and work days lost).

Regarding immune responses, QIV showed significant superiority over TIV, considering the lineage mismatch of the influenza vaccine’s B lineage. The review of Huang [18] was the only one that presented data on seroconversion and seroprotection rates for QIV compared to TIV in children and adolescents. The seroconversion rates of the QIV in the population aged between 6 months to 3 years were approximately 5 times higher (RR: 4.74 – CI95%: 2.76; 8.14), and 3 times higher in children and adolescents (3 to 18 years) (RR: 3.09 – CI95%: 1.99; 4.78) compared to TIV in a mismatch season (Appendix Table 4).

The QIV was also considered superior to the TIV for seroconversion and seroprotection rates, considering the influenza vaccine’s B lineage mismatch in adults (> 18 years and < 65 years), respectively, with relative risks ranging from 1.78 to 2.20, and 1.12 to 1.34 (Appendix Table 4).

One review [18] included data on the seroconversion and seroprotection rates of the QIV compared with the TIV in the population of children aged between 6 months to 3 years. Due to the absence of primary studies evaluating the TIV containing the B/Victoria lineage, the data available for this population showed a comparison between the QIV and TIV including the B/Yamagata. The seroconversion rate to the lineage B/Victoria was deemed to be superior in the QIV compared with the TIV including the B/Yamagata. The seroprotection rate for the B/Victoria lineage was also significantly higher for the QIV compared to the TIV including the B/Yamagata.

Considering the scenario with lineage match between the QIV vs. TIV, in four reviews [17, 18, 20, 21] no significant differences were found in seroconversion rates between QIV and TIV in seasons with no mismatch. Regarding the B/Victoria and B/Yamagata lineages, the QIV also presented no significant differences compared with the TIV.

The pooled estimate provided by Minozzi and Colleagues [52] demonstrated that both QIV and TIV provided reductions in the incidence of laboratory-confirmed influenza in all age groups. In addition, there were no significant differences between the QIV and TIV for all outcomes of interest (i.e., hospitalizations, influenza-like illness). Details on the comparisons are presented in Appendix Table 5.

### Adverse events

Data on adverse events are detailed in Table 4, Appendix Table 4, and Appendix Table 5. Overall, our findings demonstrated that the safety of QIV compared to TIV was considered similar. There were no differences between the QIV and TIV regarding the occurrence of serious or systemic adverse events in all age groups.

However, as detailed in Appendix Table 4, the QIV presented a higher occurrence of injection site pain in children and adolescents aged from 6 months to 18 years (RR: 1.09 - CI95%: 1.02;1.17) [18], and in the adult population (> 18 years) (RR: 1.23; CI95%: 1.05; 1.44) [17]. Minozzi [52] highlighted that for the elderly population, influenza vaccines were less tolerated than in adults and children. In addition, their specific findings for inactivated vaccines (Appendix Table 5) showed that the QIV presented slightly more occurrences of systemic and local adverse events compared with TIV, though no significant differences were found (OR 1.13 – CI95%: 0.97; 1.32 and OR 1.28 - CI95%: 0.91; 1.81 respectively). Moa and colleagues included only two studies including elderly individuals; however, their conclusions were limited as they were not able to summarize the data from elderly participants.

### Certainty of evidence

The summary of findings is presented in Table 4. One of the systematic reviews [52] was not included in the GRADE assessment because the authors performed a network meta-analysis composed of direct and indirect comparisons between influenza vaccination strategies. The certainty of the evidence provided by the data from 4 systematic reviews [17, 18, 20, 21] were classified, mostly, as moderate quality, with a few comparisons judged as low-quality. Details regarding the evidence profile are presented in Appendix Table 4.

**Table 4:** GRADE summary of findings table for the critical and important outcomes. Data on immunogenicity considered the Influenza’s vaccine lineage mismatch, and safety considered pooled data, as available within the included reviews

Outcomes	Anticipated absolute effects^*^ (95% CI)		Relative effect (95% CI)	№ of participants (studies)	Certainty of the evidence (GRADE)	Comments		
	**Risk with Trivalent vaccine**	**Risk with Quadrivalent vaccine**						
**Seroconversion rate (SCR) -** Comparison within lineage mismatch (QIV B/Victoria vs. TIV with B/Yamagata OR QIV B/Yamagata vs. TIV with B/Victoria)								
**Age**: 6 months to 3 years								
**HUANG 2020** QIV B/Victoria vs. TIV with B/Yamagata	104 per 1,000	**494 per 1,000** (288 to 848)	**RR 4.74** (2.76 to 8.14)	2678 (5 RCTs)	⨁⨁◯◯ Low^b,d^	-		
**Age**: 3 years to 18 years								
**HUANG 2020** QIV B/Victoria vs. TIV with B/Yamagata	248 per 1,000	**768 per 1,000** (494 to 1,000)	**RR 3.09** (1.99 to 4.78)	6628 (5 RCTs)	⨁⨁◯◯ Low^b,d^	-		
**HUANG 2020** QIV B/Yamagata vs. TIV with B/Victoria	355 per 1,000	**817 per 1,000** (650 to 1,000)	**RR 2.30** (1.83 to 2.88)	6435 (5 RCTs)	⨁⨁◯◯ Low^b b,c^	-		
**Age**: adults (> 18 years and < 65 years)								
**MOA 2016** QIV B/Victoria vs. TIV with B/Yamagata	383 per 1,000	**681 per 1,000** (474 to 975)	**RR 1.78** (1.24 to 2.55)	4342 (4 RCTs)	⨁⨁⨁◯ Moderate^b^	-		
**LIANG 2021** QIV B/Victoria vs. TIV with B/Yamagata	316 per 1,000	**696 per 1,000** (456 to 1,000)	**RR 2.20** (1.44 to 3.37)	5291 (7 RCTs)	⨁⨁⨁◯ Moderate^b^	-		
**MENG 2018** QIV B/Victoria vs. TIV with B/Yamagata	385 per 1,000	**766 per 1,000** (516 to 1,000)	**RR 1.99** (1.34 to 2.97)	4368 (6 RCTs)	⨁⨁⨁◯ Moderate^b^	-		
**MOA 2016** QIV B/Yamagata vs. TIV with B/Victoria	376 per 1,000	**793 per 1,000** (568 to 1,000)	**RR 2.11** (1.51 to 2.95)	4623 (5 RCTs)	⨁⨁⨁◯ Moderate^b^	-		
**LIANG 2021** QIV B/Yamagata vs. TIV with B/Victoria	351 per 1,000	**660 per 1,000** (537 to 810)	**RR 1.88** (1.53 to 2.31)	5257 (7 RCTs)	⨁⨁⨁◯ Moderate^b^	-		
**MENG 2018** QIV B/Yamagata vs. TIV with B/Victoria	356 per 1,000	**690 per 1,000** (533 to 889)	**RR 1.94** (1.50 to 2.50)	4404 (6 RCTs)	⨁⨁⨁◯ Moderate^b^	-		
**Seroprotection rate (SPR) – Comparison within lineage mismatch** (QIV B/Victoria vs. TIV with B/Yamagata OR QIV B/Yamagata vs. TIV with B/Victoria)								
**Age**: 6 months to 3 years								
**HUANG 2020** QIV B/Victoria vs. TIV with B/Yamagata	253 per 1,000	**611 per 1,000** (360 to 1,000)	**RR 2.41** (1.42 to 4.10)	988 (3 RCTs)	⨁⨁◯◯ Low ^b,c^	-		
**Age**: 3 years to 18 years								
**HUANG 2020** QIV B/Victoria vs. TIV with B/Yamagata	636 per 1,000	**1000 per 1,000** (776 to 1,000)	**RR 1.72** (1.22 to 2.41)	4895 (4 RCTs)	⨁⨁⨁◯ Moderate^b^	-		
**HUANG 2020** QIV B/Yamagata vs. TIV with B/Victoria	884 per 1,000	**1000 per 1,000** (911 to 1,000)	**RR 1.16** (1.03 to 1.30)	4690 (3 RCTs)	⨁⨁◯◯ Low^b,d^	-		
**Age**: adults (> 18 years and < 65 years)								
**MOA 2016** QIV B/Victoria vs. TIV with B/Yamagata	882 per 1,000	**1000 per 1,000** (909 to 1,000)	**RR 1.14** (1.03 to 1.25)	4354 (4 RCTs)	⨁⨁⨁◯ Moderate^b^	-		
**LIANG 2021** QIV B/Victoria vs. TIV with B/Yamagata	722 per 1,000	**967 per 1,000** (794 to 1,000)	**RR 1.34** (1.10 to 1.63)	5303 (7 RCTs)	⨁⨁◯◯ Low^b,c^	-		
**MENG 2018** QIV B/Victoria vs. TIV with B/Yamagata	791 per 1,000	**1000 per 1,000** (854 to 1,000)	**RR 1.28** (1.08 to 1.51)	4440 (6 RCTs)	⨁⨁◯◯ Low^b,c^	-		
**MOA 2016** QIV B/Yamagata vs. TIV with B/Victoria	907 per 1,000	**1000 per 1,000** (925 to 1,000)	**RR 1.12** (1.02 to 1.22)	4634 (5 RCTs)	⨁⨁◯◯ Low^b,c^	-		
**LIANG 2021** QIV B/Yamagata vs. TIV with B/Victoria	892 per 1,000	**990 per 1,000** (919 to 1,000)	**RR 1.11** (1.03 to 1.19)	5278 (7 RCTs)	⨁⨁⨁◯ Moderate^b^	-		
**MENG 2018** QIV B/Yamagata vs. TIV with B/Victoria	903 per 1,000	**993 per 1,000** (921 to 1,000)	**RR 1.10** (1.02 to 1.18)	4415 (6 RCTs)	⨁⨁◯◯ Low^b,c^	-		
**Safety: Adverse Events post-vaccination**								
**Solicited injection site symptoms - QIV vs. pooled TIV**								
HUANG 2020	485 per 1,000	**441 per 1,000** (354 to 553)	**RR 0.91** (0.73 to 1.14)	9311 (7 RCTs)	⨁⨁⨁◯ Moderate^b^	-		
**Solicited general symptoms - QIV vs. pooled TIV**								
HUANG 2020	420 per 1,000	**462 per 1,000** (390 to 542)	**RR 1.10** (0.93 to 1.29)	10,094 (6 RCTs)	⨁⨁◯◯ Low^a,b^	-		
**Unsolicited adverse events - QIV vs. pooled TIV**								
HUANG 2020	347 per 1,000	**358 per 1,000** (326 to 396)	**RR 1.03** (0.94 to 1.14)	15,714 (8 RCTs)	⨁⨁◯◯ Low^a,b^	-		
**Serious adverse events - QIV vs. pooled TIV**								
HUANG 2020	14 per 1,000	**13 per 1,000** (9 to 17)	**RR 0.91** (0.67 to 1.23)	15,925 (9 RCTs)	⨁⨁⨁◯ Moderate^b^	-		
**Injection site symptoms (pain) - QIV vs. pooled TIV**								
HUANG 2020	430 per 1,000	**469 per 1,000** (439 to 504)	**RR 1.09** (1.02 to 1.17)	11,113 (8 RCTs)	⨁⨁⨁◯ Moderate^b^	-		
**Injection site symptoms (fever) - QIV vs. pooled TIV**								
HUANG 2020	370 per 1,000	**392 per 1,000** (344 to 444)	**RR 1.06** (0.93 to 1.20)	8360 (6 RCTs)	⨁⨁◯◯ Low^b^	-		
**Injection site symptoms (irritability) - QIV vs. pooled**								
HUANG 2020	370 per 1,000	**355 per 1,000** (292 to 433)	**RR 0.96** (0.79 to 1.17)	7998 (5 RCTs)	⨁⨁◯◯ Low^a,b^	-		
**Local adverse events - QIV vs. pooled TIV - adults (> 18 years)**								
MOA 2016	459 per 1,000	**533 per 1,000** (441 to 643)	**RR 1.16** (0.96 to 1.40)	2344 (3 RCTs)	⨁⨁◯◯ Low^a,b^	-		
**Systemic adverse events - QIV vs. pooled TIV - adults (> 18 years)**								
MOA 2016	351 per 1,000	**376 per 1,000** (334 to 421)	**RR 1.07** (0.95 to 1.20)	2344 (3 RCTs)	⨁⨁⨁◯ Moderate^b^	-		
**Injection site pain - QIV vs. TIV with B/Victoria - adults (> 18 years)**								
MOA 2016	407 per 1,000	**464 per 1,000** (379 to 570)	**RR 1.14** (0.93 to 1.40)	6104 (4 RCTs)	⨁⨁◯◯ Low^a,b^	-		
**Injection site pain - QIV vs. TIV with B/Yamagata - adults (> 18 years)**								
MOA 2016	356 per 1,000	**438 per 1,000** (374 to 513)	**RR 1.23** (1.05 to 1.44)	5502 (3 RCTs)	⨁⨁⨁◯ Moderate^b^	-		
**Local adverse events - QIV vs. pooled TIV**								
LIANG, 2021	407 per 1,000	**452 per 1,000** (407 to 501)	**RR 1.11** (1.00 to 1.23)	5672 (6 RCTs)	⨁⨁⨁◯ Moderate^b^	-		
**Systemic adverse events - QIV vs. pooled TIV**								
LIANG, 2021	310 per 1,000	**325 per 1,000** (300 to 350)	**RR 1.05** (0.97 to 1.13)	5672 (6 RCTs)	⨁⨁⨁◯ Moderate^b^	-		

*The risk in the intervention group (and its 95% confidence interval) is based on the assumed risk in the comparison group and the **relative effect** of the intervention (and its 95% CI).

**CI**: confidence interval; **RR**: risk ratio

**GRADE Working Group grades of evidence**

**High certainty**: we are very confident that the true effect lies close to that of the estimate of the effect.

**Moderate certainty**: we are moderately confident in the effect estimate: the true effect is likely to be close to the estimate of the effect, but there is a possibility that it is substantially different.

**Low certainty**: our confidence in the effect estimate is limited: the true effect may be substantially different from the estimate of the effect.

**Very low certainty**: we have very little confidence in the effect estimate: the true effect is likely to be substantially different from the estimate of effect.

*Explanations*

a. High heterogeneity (I2 > 60% and significant chi-square), with some overlap of the 95% CI, and some differences in the direction of the effect.

b. Did not search gray literature, references or RCT registry sites, did not perform funnel plot.

c. High heterogeneity (I2 > 60% and significant chi-square), with some overlap of the 95% CI.

d. High heterogeneity (I2 > 60% and significant chi-square), with no substantial overlap of the 95% CI.

e. Not all 95% CI overlap at least one point estimate, and some differences in the direction of the effect.

## Discussion

The objective of our study was to summarize the evidence from systematic reviews that examined the immunogenicity and safety of the inactivated quadrivalent vaccine compared to the trivalent vaccine. Our results demonstrated that the seroprotection and seroconversion rates for QIV were superior to TIV in all age groups when there was a vaccine B-lineage mismatch. Additionally, the safety profiles of QIV and TIV were deemed to be similar, with no reports of serious or systemic adverse events. However, it is important to note that pain at the injection site was significantly greater for QIV. Overall, QIV is expected to enhance protection and reduce the burden of disease caused by B-lineage mismatch across all age groups [53, 54]. This perspective is particularly interesting considering the geographical variations in the duration of seasonal influenza activity and the common occurrence of co-circulation of two influenza B lineages [55, 56].

While there were no significant differences in the occurrence of local or systemic adverse events between QIV and TIV, the included systematic reviews did report some adverse events. In adults, the main local adverse reactions included redness, swelling, and pain at the injection site. Systemic events included fatigue, headache, myalgia, and fever [21]. In children, the main adverse reactions reported were diarrhea, nasopharyngitis, cough, and oropharyngeal pain. QIV was associated with a higher incidence of pain at the injection site compared to TIV, which may be attributed to the higher concentration of QIV (60 mg) compared to TIV (45 mg) [57].

The quality and risk of bias assessment revealed an overall low quality of the included systematic reviews. This was due to the absence of protocol registration, lack of a list of excluded studies after full-text reading, inadequate information on funding/sponsors, and non-comprehensive search strategies. While three studies were classified as low risk [17, 20, 21], all the included systematic reviews had issues related to the identification and selection of studies in the ROBIS assessment. The quality assessments raised concerns regarding selection bias, suggesting that important studies may have been excluded from the systematic reviews [58]. Thus, the potential for outcome selection bias should be considered due to the absence of protocol registration and lack of information on the list of excluded studies during full-text reading [59]. The evidence certainty (GRADE assessment) ranged from low to moderate, primarily due to suspected publication bias and inconsistency issues resulting from high heterogeneity. This is significant as previous studies have shown that publication and selection bias can impact resource allocation, policy decisions, and potentially lead to an overestimation of effect sizes [58–62].

From a practical standpoint, the evidence suggests with low to moderate certainty that when a vaccine B-lineage mismatch occurs, QIV is superior to TIV in children, adolescents, and adults, leading to higher seroconversion and seroprotection rates approximately 21 days after vaccination. Although there were only a few randomized trials with elderly individuals (> 60 years of age) included in two reviews [17, 52], it is expected that QIV would also elicit a better immunogenicity response in this population. However, caution is recommended, and further high-quality trials with elderly individuals are needed. The evidence also indicates that QIV and TIV have a similar occurrence of solicited and unsolicited systemic adverse events, with no reports of serious adverse events. However, individuals may experience more pain at the injection site approximately 7 days after QIV vaccination.

We also observed that the reviews did not consider the influence of the fabrication method (split or subunit) on their results, despite 77% of the primary studies using a split vaccine, 13% using subunit vaccines, and 10% of the studies not reporting the type of vaccine. This is noteworthy because the fabrication method can impact the effects and immunogenicity [63–69]. For example, subunit influenza vaccines have certain disadvantages such as relatively low immunogenicity, higher vaccine doses required, and higher manufacturing costs [70]. On the other hand, split influenza vaccines have been considered safer for achieving effective immunization against influenza [66]. Therefore, future studies should consider a detailed description of the type of vaccine investigated, including the use of adjuvants or high doses. Moreover, subgroup analysis is warranted to investigate whether the fabrication method influences the effectiveness and occurrence of local and systemic adverse events in all age groups.

### Strengths and limitations

This overview made efforts to minimize bias by having at least two overview authors independently assess the studies for inclusion and carry out data extraction. Additionally, two independent reviewers performed quality assessments using AMSTAR 2, ROBIS, and GRADE. Furthermore, we implemented a comprehensive search strategy that encompassed major databases without language or date restrictions.

One aspect that warrants consideration in the findings of this overview is the potential presence of selection bias in 4 out of the 5 included studies. This bias arises from the lack of a registered protocol and the high risk of bias associated with the search strategy employed. This aspect may have limited our summary of the immunogenicity and safety of the QIV. However, it is unlikely that the estimates and direction of the effect would change to a significant extent, even though important primary studies may have been omitted from the systematic reviews. Another limitation pertains to our conclusions regarding vaccination clinical efficacy, specifically the capacity to prevent infections [71]. While we planned to include outcomes related to clinical efficacy (e.g., hospitalization, mortality), we were unable to summarize this data. It is worth noting that there is a challenge in translating immunogenicity to disease burden, as immune responses after vaccination do not always accurately predict real protection against a disease [72].

## Conclusion

Our findings indicate that QIV elicits a superior immunogenicity response compared to TIV in all age groups evaluated, particularly in the presence of a lineage mismatch. The safety of QIV is similar to TIV, with no reports of serious or systemic adverse events. However, there is a greater incidence of pain at the injection site with QIV. We advise caution due to the high risk of bias in the selection process and the lack of protocol registration. This overview recommends that researchers provide clearer information regarding the identification and selection of studies, as well as prospective protocol registration.

### Electronic supplementary material

Below is the link to the electronic supplementary material.

Supplementary Material 1

## Data Availability

All relevant data are within the paper and its Supporting Information files.
