# Supplementary material for: Immunogenicity and safety of inactivated quadrivalent influenza vaccine compared with the trivalent vaccine for influenza infection: an overview of systematic reviews
Source: BMC Infect Dis. 2023 Aug 29;23:563. doi: 10.1186/s12879-023-08541-0 (PMC10463610; doi:10.1186/s12879-023-08541-0)
Supplement: Supplementary file 1 — Supplementary Material 1 [file 12879_2023_8541_MOESM1_ESM.docx]

**Appendix Table 1.** Search strategy adopted in the study.

| **Database** | **Search strategy** | **Results** |
| --- | --- | --- |
| **MEDLINE via PUBMED** | (((((grippe[Title/Abstract]) OR (influenza[Title/Abstract])) OR (flu[Title/Abstract])) AND (((vaccine[Title/Abstract]) OR (immunization[Title/Abstract])) OR (vaccination[Title/Abstract]))) AND (trivalent[Title/Abstract])) AND ((quadrivalent[Title/Abstract]) OR (tetravalent[Title/Abstract])) | **314** |
| **COCHRANE** | (("influenza"):ti,ab,kw OR ("flu"):ti,ab,kw OR ("grippe"):ti,ab,kw) AND ("vaccine" or "immunization") AND ("Trivalent") AND ("Quadrivalent" or "Tetravalent") | **141** |
| **EMBASE** | grippe:ti,ab,kw OR flu:ti,ab,kw OR influenza:ti,ab,kw AND vaccine:ti,ab,kw OR immunization:ti,ab,kw OR vaccination:ti,ab,kw AND trivalent:ti,ab,kw AND tetravalent:ti,ab,kw OR quadrivalent: ti,ab,kw | **397** |
| **LILACS** | Influenza OR flu OR grippe [Words] AND vaccine OR immunization OR vaccination [Words] and trivalent [Words] and quadrivalent OR Tetravalent [Words] | **309** |
| **SCOPUS** | (TITLE-ABS-KEY (influenza) OR TITLE-ABS-KEY (flu) OR TITLE-ABS-KEY (grippe) AND TITLE-ABS-KEY (vaccine) OR TITLE-ABS-KEY (immunization) OR TITLE-ABS-KEY (vaccination) AND TITLE-ABS-KEY (tetravalent) OR TITLE-ABS-KEY (quadrivalent) AND TITLE-ABS-KEY (trivalent)) | **339** |
| **Web of**  **Science** | ALL=(influenza) OR ALL=(grippe) OR ALL=(flu) AND ALL=(vaccine) OR ALL=(vaccination) OR ALL=(immunization) AND ALL=(trivalent) AND ALL=(tetravalent) OR ALL=(quadrivalent) | **319** |
| **CINAHL (EBSCO)** | (("influenza") OR ("flu") OR ("grippe")) AND ("vaccine" or "immunization" or "vaccination”) AND ("Trivalent") AND ("Quadrivalent" or "Tetravalent") | **71** |
| **TOTAL** | | **1,890** |

**Appendix Table 2**. List of excluded studies, with reasons for exclusion after full text reading.

| **Author** | **Study** | **Design** | **Reason for exclusion** |
| --- | --- | --- | --- |
| Barr and Jelley ^1^ | The Coming Era of Quadrivalent Human Influenza Vaccines: Who will Benefit? | Narrative review | Wrong study design, does not compare the efficacy of the quadrivalent inactivated vaccines vs trivalent inactivated vaccines |
| Mckenzie ^2^ | Why Get a Flu Shot? | Press release | Wrong study design, does not compare the efficacy of the quadrivalent inactivated vaccines vs trivalent inactivated vaccines |
| Gemmill ^3^ | Summary of the National Advisory Committee on Immunization (NACI) Statement on Seasonal Influenza Vaccine for 2015-2016. | Narrative review | Wrong study design, does not compare the efficacy of the quadrivalent inactivated vaccines vs trivalent inactivated vaccines |
| Moore ^4^ | Vaccine recommendations for children and youth for the 2015/2016 influenza season. | Narrative review | Wrong study design, does not compare the efficacy of the quadrivalent inactivated vaccines vs trivalent inactivated vaccines |
| Beyer, et al. ^5^ | Rationale for two influenza B lineages in seasonal vaccines: A meta-regression study on immunogenicity and controlled field trials | Systematic Review | Presents data related to a meta regression, and does not compare the efficacy of the quadrivalent vs trivalent vaccine |
| Caspard, et al. ^6^ | Live-Attenuated Influenza Vaccine Effectiveness in Children From 2009 to 2015-2016: A Systematic Review and Meta-Analysis | Systematic Review and Meta-Analysis | Inadequate interventions, only evaluates live-attenuated influenza vaccine |
| Grohskopf, et al. ^7^ | Prevention and Control of Seasonal Influenza with Vaccines: recommendations of the Advisory Committee on Immunization Practices--United States, 2018-19 Influenza Season. | Narrative review | Wrong study design, does not compare the efficacy of the quadrivalent inactivated vaccines vs trivalent inactivated vaccines |
| Castro, et al. ^8^ | Seasonal influenza immunisation for older adults in Australia: vaccine options for 2019. | Narrative review | Wrong study design, does not compare the efficacy of the quadrivalent inactivated vaccines vs trivalent inactivated vaccines |
| Sinilaite, et al. ^9^ | Summary of the NACI Supplemental Statement on Mammalian Cell Culture-Based Influenza Vaccines. | Rapid review | Presents no data on the efficacy and safety, but only recommendations from the National advisory committee on immunization of Canada |
| Gaglani and Flannery ^10^ | Reply to Yager and Doll | Letter to the editor | Wrong study design, does not compare the efficacy of the quadrivalent inactivated vaccines vs trivalent inactivated vaccines |
| Yager and Doll ^11^ | Towards Understanding the Health and Economic Impacts of Quadrivalent and Trivalent Inactivated Vaccines Against Influenza B Infection: Additional Considerations for Future Cost-Benefit Analyses | Letter to the editor | Wrong study design, does not compare the efficacy of the quadrivalent inactivated vaccines vs trivalent inactivated vaccines |
| Meyer, et al. ^12^ | Vaccination against influenza saves lives - a 2021 update. | Narrative review | Wrong study design, does not compare the efficacy of the quadrivalent inactivated vaccines vs trivalent inactivated vaccines |
| Coleman, et al. ^13^ | Effectiveness of the MF59-adjuvanted trivalent or quadrivalent seasonal influenza vaccine among adults 65 years of age or older, a systematic review and meta-analysis | Systematic Review and Meta-Analysis | Inadequate interventions, evaluated only trivalent and quadrivalent inactivated vaccines with squalene-based adjuvant MF59® |
| Sinilaite, et al. ^14^ | Summary of the National Advisory Committee on Immunization (NACI) Seasonal Influenza Vaccine Statement for 2021-2022. | Narrative review | Wrong study design, does not compare the efficacy of the quadrivalent inactivated vaccines vs trivalent inactivated vaccines |
| JAMA ^15^ | Influenza Vaccine for 2019-2020. | Narrative review | Wrong study design, does not compare the efficacy of the quadrivalent inactivated vaccines vs trivalent inactivated vaccines |
| Lockwood ^16^ | How flu shots help protect the public: Here's what ob/gyns need to know when treating their at-risk patients. | Special report | Wrong study design, does not compare the efficacy of the quadrivalent inactivated vaccines vs trivalent inactivated vaccines |
| No author ^17^ | Ample vaccine supply key to combatting unpredictability of flu season. | Press release from Drug Topics | Wrong study design, does not compare the efficacy of the quadrivalent inactivated vaccines vs trivalent inactivated vaccines |
| No author ^18^ | Bracing for flu season: Steps to protect yourself right now. | Press release from Harvard Medical School | Wrong study design, does not compare the efficacy of the quadrivalent inactivated vaccines vs trivalent inactivated vaccines |
| Lindsey, et al. ^19^ | The efficacy, effectiveness, and immunogenicity of influenza vaccines in Africa: a systematic review | Systematic review | Presents data related to trivalent inactivated vaccine, and live attenuated vaccine, but does not compare the efficacy of the quadrivalent inactivated vaccines vs trivalent inactivated vaccines |

**References of the excluded studies**

1. Barr IG, Jelley LL. The coming era of quadrivalent human influenza vaccines: who will benefit? Drugs 2012; 72:2177-85.

2. Mckenzie L. Why Get a Flu Shot? . Phoenix, Arizona: PVA Publications - a division of Paralyzed Veterans of America, Inc, 2015:22-3.

3. Gemmill I. Summary of the National Advisory Committee on Immunization (NACI) Statement on Seasonal Influenza Vaccine for 2015-2016. Can Commun Dis Rep 2015; 41:227-32.

4. Moore DL. Vaccine recommendations for children and youth for the 2015/2016 influenza season. Paediatr Child Health 2015; 20:389-94.

5. Beyer WEP, Palache AM, Boulfich M, Osterhaus A. Rationale for two influenza B lineages in seasonal vaccines: A meta-regression study on immunogenicity and controlled field trials. Vaccine 2017; 35:4167-76.

6. Caspard H, Mallory RM, Yu J, Ambrose CS. Live-Attenuated Influenza Vaccine Effectiveness in Children From 2009 to 2015-2016: A Systematic Review and Meta-Analysis. Open Forum Infect Dis 2017; 4:ofx111.

7. Grohskopf LA, Sokolow LZ, Broder KR, Walter EB, Fry AM, Jernigan DB. Prevention and Control of Seasonal Influenza with Vaccines: Recommendations of the Advisory Committee on Immunization Practices-United States, 2018-19 Influenza Season. MMWR Recomm Rep 2018; 67:1-20.

8. Castro Md, Leeb A, Buynder PV. Seasonal influenza immunisation for older adults in Australia: Vaccine options for 2019: Australian Nursing and Midwifery Federation; 2019.

9. Sinilaite A, Gemmill I, Harrison R. Summary of the NACI Supplemental Statement on Mammalian Cell Culture-Based Influenza Vaccines. Can Commun Dis Rep 2020; 46:324-32.

10. Gaglani M, Flannery B. Reply to Yager and Doll. Clinical Infectious Diseases 2020; 71:3264-5.

11. Yager EJ, Doll MK. Towards Understanding the Health and Economic Impacts of Quadrivalent and Trivalent Inactivated Vaccines Against Influenza B Infection: Additional Considerations for Future Cost-Benefit Analyses. Clin Infect Dis 2020; 71:3263-4.

12. Meyer J, Sibanda M, Burnett R. Vaccination against influenza saves lives – a 2021 update. Professional Nursing Today 2021; 25:5.

13. Coleman BL, Sanderson R, Haag MDM, McGovern I. Effectiveness of the MF59-adjuvanted trivalent or quadrivalent seasonal influenza vaccine among adults 65 years of age or older, a systematic review and meta-analysis. Influenza Other Respir Viruses 2021; 15:813-23.

14. Sinilaite A, Young K, Harrington R. Summary of the National Advisory Committee on Immunization (NACI) Seasonal Influenza Vaccine Statement for 2021-2022. Can Commun Dis Rep 2021; 47:372-80.

15. JAMA. Influenza Vaccine for 2019-2020. JAMA 2020; 323:84-5.

16. Lockwood CJ, (Editor). How flu shots help protect the public. Here’s what ob/gyns need to know when treating their at-risk patients. Contemporary OB/GYN 2019; 64:2.

17. NA. Ample vaccine supply key to combating unpredictability of flu season. Drug Topics, 2013.

18. NA. Bracing for flu season: Steps to protect yourself right now. Harvard Medical School, 2021.

19. Lindsey BB, Armitage EP, Kampmann B, de Silva TI. The efficacy, effectiveness, and immunogenicity of influenza vaccines in Africa: a systematic review. Lancet Infect Dis 2019; 19:e110-e9.

**Appendix Table 3.** Assessment of the methodological quality (AMSTAR 2) of the included systematic reviews.

| **AMSTAR item** | **Moa 2016** | **Meng 2018** | **Huang 2020** | **Liang 2021** | **Minozzi 2022** |
| --- | --- | --- | --- | --- | --- |
| 1) Did the research questions and inclusion criteria for the review include the components of PICO? | Yes | Yes | Yes | Yes | Yes |
| 2) Did the report of the review contain an explicit statement that the review methods were established prior to the conduct of the review and did the report justify any significant deviations from the protocol? | No | No | No | No | Yes |
| 3) Did the review authors explain their selection of the study designs for inclusion in the review? | Yes | Yes | Yes | Yes | Yes |
| 4) Did the review authors use a comprehensive literature search strategy? | Partial Yes | Partial Yes | Partial Yes | Partial Yes | Partial Yes |
| 5) Did the review authors perform study selection in duplicate? | Yes | Yes | Yes | Yes | Yes |
| 6) Did the review authors perform data extraction in duplicate? | Yes | Yes | Yes | Yes | Yes |
| 7) Did the review authors provide a list of excluded studies and justify the exclusions? | No | No | No | No | Yes |
| 8) Did the review authors describe the included studies in adequate detail? | Yes | Partial Yes | Yes | Partial Yes | Partial Yes |
| 9) RCTs: Did the review authors use a satisfactory technique for assessing the risk of bias (RoB) in individual studies that were included in the review? | Yes | Yes | Yes | Yes | Yes |
| 10) Did the review authors report on the sources of funding for the studies included in the review? | No | No | No | No | Yes |
| 11) RCTs: If meta-analysis was performed did the review authors use appropriate methods for statistical combination of results? | Yes | Yes | Yes | Yes | Yes |
| 12) If meta-analysis was performed, did the review authors assess the potential impact of RoB in individual studies on the results of the meta-analysis or other evidence synthesis? | No | No | No | No | No |
| 13) Did the review authors account for RoB in individual studies when interpreting/ discussing the results of the review? | Yes | No | No | No | Yes |
| 14) Did the review authors provide a satisfactory explanation for, and discussion of, any heterogeneity observed in the results of the review? | Yes | Yes | Yes | Yes | Yes |
| 15) If they performed quantitative synthesis did the review authors carry out an adequate investigation of publication bias (small study bias) and discuss its likely impact on the results of the review? | No | No | No | No | No |
| 16) Did the review authors report any potential sources of conflict of interest, including any funding they received for conducting the review? | Yes | Yes | Yes | Yes | Yes |
| ROB: Cochrane’s Risk of Bias tool; RCTs: Randomized Controlled Trials | | | | | |

**Appendix Table 4.** GRADE evidence profile table (critical and important outcomes).

| **Certainty assessment** | | | | | | | **№ of patients** | | **Effect** | | **Certainty** | **Importance** |
| --- | --- | --- | --- | --- | --- | --- | --- | --- | --- | --- | --- | --- |
| **№ of studies** | **Study design** | **Risk of bias** | **Inconsistency** | **Indirectness** | **Imprecision** | **Other considerations** | **Quadrivalent vaccine** | **Trivalent vaccine** | **Relative (95% CI)** | **Absolute (95% CI)** |  |  |
| **Seroconversion rate (SCR) - Comparison within lineage mismatch** (QIV B/Victoria vs TIV with B/Yamagata OR QIV B/Yamagata vs TIV with B/Victoria) | | | | | | | | | | | | |
| **Age: 6 months to 3 years** (follow-up: mean 21 days; assessed with: number of participants) | | | | | | | | | | | | |
| **HUANG 2020**  B/Victoria vs TIV with B/Yamagata | 5 randomised trials | not serious | serious^d^ | not serious | not serious | publication bias strongly suspected^b^ | 1061/1824 (58.2%) | 89/854 (10.4%) | **RR 4.74** (2.76 to 8.14) | **390 more per 1,000** (from 183 more to 744 more) | ⨁⨁◯◯ Low | IMPORTANT |
| **Age: 3 years to 18 years** (follow-up: mean 21 days; assessed with: number of participants) | | | | | | | | | | | | |
| **HUANG 2020**  QIV B/Victoria vs TIV with B/Yamagata | 5 randomised trials | not serious | serious^d^ | not serious | not serious | publication bias strongly suspected^b^ | 2966/4108 (72.2%) | 626/2520 (24.8%) | **RR 3.09** (1.99 to 4.78) | **519 more per 1,000** (from 246 more to 939 more) | ⨁⨁◯◯ Low | IMPORTANT |
| **HUANG 2020**  QIV B/Yamagata vs TIV with B/Victoria | 5 randomised trials | not serious | serious^c^ | not serious | not serious | publication bias strongly suspected^b^ | 2973/3945 (75.4%) | 884/2490 (35.5%) | **RR 2.30** (1.83 to 2.88) | **462 more per 1,000** (from 295 more to 667 more) | ⨁⨁◯◯ Low | IMPORTANT |
| **Age: adults (>18 years and <65 years)** (follow-up: mean 21 days; assessed with: number of participants) | | | | | | | | | | | | |
| **MOA 2016**  QIV B/Victoria vs TIV with B/Yamagata | 4 randomised trials | not serious | not serious | not serious | not serious | publication bias strongly suspected^b^ | 1924/3359 (57.3%) | 376/983 (38.3%) | **RR 1.78** (1.24 to 2.55) | **298 more per 1,000** (from 92 more to 593 more) | ⨁⨁⨁◯ Moderate | IMPORTANT |
| **MOA 2016**  QIV B/Yamagata vs TIV with B/Victoria | 5 randomised trials | not serious | not serious | not serious | not serious | publication bias strongly suspected^b^ | 2186/3463 (63.1%) | 436/1160 (37.6%) | **RR 2.11** (1.51 to 2.95) | **417 more per 1,000** (from 192 more to 733 more) | ⨁⨁⨁◯ Moderate | IMPORTANT |
| **LIANG 2021**  QIV B/Victoria vs TIV with B/Yamagata | 7 randomised trials | not serious | not serious | not serious | not serious | publication bias strongly suspected^b^ | 2318/3907 (59.3%) | 438/1384 (31.6%) | **RR 2.20** (1.44 to 3.37) | **380 more per 1,000** (from 139 more to 750 more) | ⨁⨁⨁◯ Moderate | IMPORTANT |
| **LIANG 2021**  QIV B/Yamagata vs TIV with B/Victoria | 7 randomised trials | not serious | not serious | not serious | not serious | publication bias strongly suspected^b^ | 2448/3812 (64.2%) | 507/1445 (35.1%) | **RR 1.88** (1.53 to 2.31) | **309 more per 1,000** (from 186 more to 460 more) | ⨁⨁⨁◯ Moderate | IMPORTANT |
| **MENG 2018**  QIV B/Victoria vs TIV with B/Yamagata | 6 randomised trials | not serious | not serious | not serious | not serious | publication bias strongly suspected^b^ | 2014/3332 (60.4%) | 399/1036 (38.5%) | **RR 1.99** (1.34 to 2.97) | **381 more per 1,000** (from 131 more to 759 more) | ⨁⨁⨁◯ Moderate | IMPORTANT |
| **MENG 2018**  QIV B/Yamagata vs TIV with B/Victoria | 6 randomised trials | not serious | not serious | not serious | not serious | publication bias strongly suspected^b^ | 2139/3237 (66.1%) | 415/1167 (35.6%) | **RR 1.94** (1.50 to 2.50) | **334 more per 1,000** (from 178 more to 533 more) | ⨁⨁⨁◯ Moderate | IMPORTANT |
| **Seroprotection rate (SPR) – Comparison within lineage mismatch** (QIV B/Victoria vs TIV with B/Yamagata OR QIV B/Yamagata vs TIV with B/Victoria) | | | | | | | | | | | | |
| **Age: 6 months to 3 years** (follow-up: mean 21 days; assessed with: number of participants) | | | | | | | | | | | | |
| **HUANG 2020**  QIV B/Victoria vs TIV with B/Yamagata | 3 randomised trials | not serious | serious^c^ | not serious | not serious | publication bias strongly suspected^b^ | 387/538 (71.9%) | 114/450 (25.3%) | **RR 2.41** (1.42 to 4.10) | **357 more per 1,000** (from 106 more to 785 more) | ⨁⨁◯◯ Low | IMPORTANT |
| **Age: 3 years to 18 years** (follow-up: mean 21 days; assessed with: number of participants) | | | | | | | | | | | | |
| **HUANG 2020**  QIV B/Victoria vs TIV with B/Yamagata | 4 randomised trials | not serious | not serious | not serious | not serious | publication bias strongly suspected^b^ | 2448/2728 (89.7%) | 1378/2167 (63.6%) | **RR 1.72** (1.22 to 2.41) | **458 more per 1,000** (from 140 more to 897 more) | ⨁⨁⨁◯ Moderate | IMPORTANT |
| **HUANG 2020**  QIV B/Yamagata vs TIV with B/Victoria | 3 randomised trials | not serious | serious^d^ | not serious | not serious | publication bias strongly suspected^b^ | 2488/2556 (97.3%) | 1887/2134 (88.4%) | **RR 1.16** (1.03 to 1.30) | **141 more per 1,000** (from 27 more to 265 more) | ⨁⨁◯◯ Low | IMPORTANT |
| **Age: adults (>18 years and <65 years)** (follow-up: mean 21 days; assessed with: number of participants) | | | | | | | | | | | | |
| **MOA 2016**  QIV B/Victoria vs TIV with B/Yamagata | 4 randomised trials | not serious | not serious | not serious | not serious | publication bias strongly suspected^b^ | 3296/3367 (97.9%) | 871/987 (88.2%) | **RR 1.14** (1.03 to 1.25) | **124 more per 1,000** (from 26 more to 221 more) | ⨁⨁⨁◯ Moderate | IMPORTANT |
| **MOA 2016**  QIV B/Yamagata vs TIV with B/Victoria | 5 randomised trials | not serious | serious^c^ | not serious | not serious | publication bias strongly suspected^b^ | 3436/3471 (99.0%) | 1055/1163 (90.7%) | **RR 1.12** (1.02 to 1.22) | **109 more per 1,000** (from 18 more to 200 more) | ⨁⨁◯◯ Low | IMPORTANT |
| **LIANG 2021**  QIV B/Victoria vs TIV with B/Yamagata | 7 randomised trials | not serious | serious^c^ | not serious | not serious | publication bias strongly suspected^b^ | 3634/3915 (92.8%) | 1002/1388 (72.2%) | **RR 1.34** (1.10 to 1.63) | **245 more per 1,000** (from 72 more to 455 more) | ⨁⨁◯◯ Low | IMPORTANT |
| **LIANG 2021**  QIV B/Yamagata vs TIV with B/Victoria | 7 randomised trials | not serious | not serious | not serious | not serious | publication bias strongly suspected^b^ | 3733/3820 (97.7%) | 1301/1458 (89.2%) | **RR 1.11** (1.03 to 1.19) | **98 more per 1,000** (from 27 more to 170 more) | ⨁⨁⨁◯ Moderate | IMPORTANT |
| **MENG 2018**  QIV B/Victoria vs TIV with B/Yamagata | 6 randomised trials | not serious | serious^c^ | not serious | not serious | publication bias strongly suspected^b^ | 3200/3340 (95.8%) | 870/1100 (79.1%) | **RR 1.28** (1.08 to 1.51) | **221 more per 1,000** (from 63 more to 403 more) | ⨁⨁◯◯ Low | IMPORTANT |
| **MENG 2018**  QIV B/Yamagata vs TIV with B/Victoria | 6 randomised trials | not serious | serious^c^ | not serious | not serious | publication bias strongly suspected^b^ | 3200/3245 (98.6%) | 1056/1170 (90.3%) | **RR 1.10** (1.02 to 1.18) | **90 more per 1,000** (from 18 more to 162 more) | ⨁⨁◯◯ Low | IMPORTANT |
| **Safety: Adverse Events post-vaccination** | | | | | | | | | | | | |
| **Solicited injection site symptoms - QIV vs pooled TIV (age: >6 months to 18 years)** (follow-up: mean 7 days; assessed with: number of participants) | | | | | | | | | | | | |
| HUANG 2020 | 7 randomised trials | not serious | not serious | not serious | not serious | publication bias strongly suspected^b^ | 3077/6338 (48.5%) | 1442/2973 (48.5%) | **RR 0.91** (0.73 to 1.14) | **44 fewer per 1,000** (from 131 fewer to 68 more) | ⨁⨁⨁◯ Moderate | CRITICAL |
| **Solicited general symptoms - QIV vs pooled TIV (age: >6 months to 18 years)** (follow-up: mean 7 days; assessed with: number of participants) | | | | | | | | | | | | |
| HUANG 2020 | 6 randomised trials | not serious | serious^a^ | not serious | not serious | publication bias strongly suspected^b^ | 3170/6705 (47.3%) | 1423/3389 (42.0%) | **RR 1.10** (0.93 to 1.29) | **42 more per 1,000** (from 29 fewer to 122 more) | ⨁⨁◯◯ Low | CRITICAL |
| **Unsolicited adverse events - QIV vs pooled TIV (age: >6 months to 18 years)** (follow-up: mean 28 days; assessed with: number of participants) | | | | | | | | | | | | |
| HUANG 2020 | 8 randomised trials | not serious | serious^a^ | not serious | not serious | publication bias strongly suspected^b^ | 3578/8836 (40.5%) | 2388/6878 (34.7%) | **RR 1.03** (0.94 to 1.14) | **10 more per 1,000** (from 21 fewer to 49 more) | ⨁⨁◯◯ Low | CRITICAL |
| **Serious adverse events - QIV vs pooled TIV (age: >6 months to 18 years)** (follow-up: mean 7 days; assessed with: number of participants) | | | | | | | | | | | | |
| HUANG 2020 | 9 randomised trials | not serious | not serious | not serious | not serious | publication bias strongly suspected^b^ | 173/9045 (1.9%) | 97/6880 (1.4%) | **RR 0.91** (0.67 to 1.23) | **1 fewer per 1,000** (from 5 fewer to 3 more) | ⨁⨁⨁◯ Moderate | CRITICAL |
| **Injection site symptoms (pain) - QIV vs pooled TIV (age: >6 months to 18 years)** (follow-up: mean 7 days; assessed with: number of participants) | | | | | | | | | | | | |
| HUANG 2020 | 8 randomised trials | not serious | not serious | not serious | not serious | publication bias strongly suspected^b^ | 3594/7236 (49.7%) | 1669/3877 (43.0%) | **RR 1.09** (1.02 to 1.17) | **39 more per 1,000** (from 9 more to 73 more) | ⨁⨁⨁◯ Moderate | CRITICAL |
| **Injection site symptoms (fever) - QIV vs pooled TIV (age: >6 months to 18 years)** (follow-up: mean 7 days; assessed with: number of participants) | | | | | | | | | | | | |
| HUANG 2020 | 6 randomised trials | not serious | not serious | not serious | serious | publication bias strongly suspected^b^ | 791/5389 (14.7%) | 1099/2971 (37.0%) | **RR 1.06** (0.93 to 1.20) | **22 more per 1,000** (from 26 fewer to 74 more) | ⨁⨁◯◯ Low | CRITICAL |
| **Injection site symptoms (irritability) - QIV vs pooled TIV (age: >6 months to 18 years)** (follow-up: mean 7 days; assessed with: number of participants) | | | | | | | | | | | | |
| HUANG 2020 | 5 randomised trials | not serious | serious^a^ | not serious | not serious | publication bias strongly suspected^b^ | 2106/5027 (41.9%) | 1099/2971 (37.0%) | **RR 0.96** (0.79 to 1.17) | **15 fewer per 1,000** (from 78 fewer to 63 more) | ⨁⨁◯◯ Low | CRITICAL |
| **Local adverse events - QIV vs pooled TIV - adults (> 18 years)** (follow-up: mean 7 days; assessed with: number of participants) | | | | | | | | | | | | |
| MOA 2016 | 3 randomised trials | not serious | serious^a^ | not serious | not serious | publication bias strongly suspected^b^ | 706/1410 (50.1%) | 429/934 (45.9%) | **RR 1.16** (0.96 to 1.40) | **73 more per 1,000** (from 18 fewer to 184 more) | ⨁⨁◯◯ Low | CRITICAL |
| **Systemic adverse events - QIV vs pooled TIV - adults (> 18 years)** (follow-up: mean 7 days; assessed with: number of participants) | | | | | | | | | | | | |
| MOA 2016 | 3 randomised trials | not serious | not serious | not serious | not serious | publication bias strongly suspected^b^ | 536/1410 (38.0%) | 328/934 (35.1%) | **RR 1.07** (0.95 to 1.20) | **25 more per 1,000** (from 18 fewer to 70 more) | ⨁⨁⨁◯ Moderate | CRITICAL |
| **Injection site pain - QIV vs TIV with B/Victoria - adults (> 18 years)** (follow-up: mean 7 days; assessed with: number of participants) | | | | | | | | | | | | |
| MOA 2016 | 4 randomised trials | not serious | serious^a^ | not serious | not serious | publication bias strongly suspected^b^ | 2021/4591 (44.0%) | 616/1513 (40.7%) | **RR 1.14** (0.93 to 1.40) | **57 more per 1,000** (from 28 fewer to 163 more) | ⨁⨁◯◯ Low | CRITICAL |
| **Injection site pain - QIV vs TIV with B/Yamagata - adults (> 18 years)** (follow-up: mean 7 days; assessed with: number of participants) | | | | | | | | | | | | |
| MOA 2016 | 3 randomised trials | not serious | not serious | not serious | not serious | publication bias strongly suspected^b^ | 1945/4486 (43.4%) | 362/1016 (35.6%) | **RR 1.23** (1.05 to 1.44) | **82 more per 1,000** (from 18 more to 157 more) | ⨁⨁⨁◯ Moderate | CRITICAL |
| **Local adverse events - QIV vs pooled TIV** (follow-up: mean 7 days; assessed with: number of participants) | | | | | | | | | | | | |
| LIANG 2021 | 6 randomised trials | not serious | not serious | not serious | not serious | publication bias strongly suspected^b^ | 1594/3349 (47.6%) | 946/2323 (40.7%) | **RR 1.11** (1.00 to 1.23) | **45 more per 1,000** (from 0 fewer to 94 more) | ⨁⨁⨁◯ Moderate | CRITICAL |
| **Systemic adverse events - QIV vs pooled TIV** (follow-up: mean 7 days; assessed with: number of participants) | | | | | | | | | | | | |
| LIANG 2021 | 6 randomised trials | not serious | not serious | not serious | not serious | publication bias strongly suspected^b^ | 1173/3349 (35.0%) | 719/2323 (31.0%) | **RR 1.05** (0.97 to 1.13) | **15 more per 1,000** (from 9 fewer to 40 more) | ⨁⨁⨁◯ Moderate | CRITICAL |

**CI:** confidence interval; **RR:** risk ratio

#### Explanations

a. High heterogeneity (I2 > 60% and significant chi-square), with some overlap of the 95% CI, and some differences in the direction of the effect

b. Did not search gray literature, references or RCT registry sites, did not perform funnel plot

c. High heterogeneity (I2 > 60% and significant chi-square), with some overlap of the 95% CI

d. High heterogeneity (I2 > 60% and significant chi-square), with no substantial overlap of the 95% CI.

e. Not all 95% CI overlap at least one point estimate, and some differences in the direction of the effect

**Appendix 5.** Data pertaining to specific comparisons between quadrivalent inactivated vaccines vs trivalent inactivated vaccines from the network metanalysis by Minozzi and Colleagues. We thank Miss Minozzi and Mr. Lytras for providing this information.

| **Outcomes** | **Comparison QIV vs TIV** |
| --- | --- |
|  | **RR (95% CrI)** |
| **Adults (18-60 years):** |  |
| Laboratory-confirmed influenza | 1.41 (0.90 - 2.15) |
| Any systemic AE | 1.02 (0.95 - 1.10) |
| Any local AE | 1.13 (0.95 - 1.35) |
| Influenza-like illness | 0.93 (0.61 - 1.41) |
| Hospitalization | 1.00 (0.28 - 3.23) |
| **Elderly (> 61 years):** |  |
| Laboratory-confirmed influenza | 0.68 (0.18 - 2.27) |
| Any systemic AE | 1.13 (0.97 - 1.32) |
| Any local AE | 1.28 (0.91 - 1.81) |
| Influenza-like illness | 2.15 (0.40 - 11.07) |
| Hospitalization | 2.32 (0.17 - 35.31) |
| **Children (< 18 years):** |  |
| Laboratory-confirmed influenza | 0.90 (0.35 - 2.40) |
| Any systemic AE | 0.98 (0.87 - 1.12) |
| Any local AE | 1.05 (0.90 - 1.22) |
| Acute Otitis Media in children | 0.59 (0.01 - 93.54) |
| Influenza-like illness | 1.57 (0.70 - 3.66) |
| Hospitalization | 0.59 (0.08 - 3.57) |
| **Young Children (up to 5 years):** |  |
| Laboratory-confirmed influenza | 0.80 (0.27 - 2.44) |
| Any systemic AE | 0.96 (0.79 - 1.19) |
| Any local AE | 1.04 (0.84 - 1.28) |
| Acute Otitis Media in children | 0.58 (0.01 - 106.19) |
| Influenza-like illness | 1.56 (0.57 - 4.49) |
| Hospitalization | 0.57 (0.07 - 3.74) |
| AE: Adverse events; 95% CrI: 95% credible interval. | |
